# PReS-FINAL-2159: Tocilizumab (TCZ) dosing in juvenile idiopathic arthritis (JIA): optimising for different JIA type and body weight patients

**DOI:** 10.1186/1546-0096-11-S2-P171

**Published:** 2013-12-05

**Authors:** P Lu, J Hsu, C Keane, S Fettner, J Wang, N Ruperto, O Harari, HI Brunner, F De Benedetti

**Affiliations:** 1 Roche, Nutley, NJ, USA; 2 Roche, Welwyn, UK; 3 PRINTO, Genoa, Italy; 4 PRCSG, Cincinnati, USA; 5 IRCCS Ospedale Ped Bambino Gesú, Rome, Italy

## Introduction

TCZ, an IL-6R inhibitor, is effective in systemic and polyarticular juvenile idiopathic arthritis (sjia, pjia). BW-adjusted, intravenous dosing regimens (TCZ8 mg/kg Q2W for sjia and Q4W for pjia) were assessed in Japanese phase 3 trials. As shown in Results, BW adjustment led to lower TCZ exposure with lower BW; thus, higher doses were proposed for patients (pts) with BW<30 kg in the global TENDER (sjia) and CHERISH (pjia) trials.

## Objectives

To describe the PK, PD and exposure-efficacy/-safety relationships of adjusted BW-based TCZ therapy in sjia/pjia pts.

## Methods

PK/PD results were summarised in TCZ-treated pts from part 1 of TENDER and CHERISH. TENDER part 1 (n = 75) comprised a 12-wk, double-blind phase with pts randomised 1:1 to TCZ or placebo Q2W; TCZ 12 mg/kg if <30 kg and 8 mg/kg if ≥30 kg. CHERISH part 1 (n = 177) comprised a 16-wk TCZ Q4W open-label phase (TCZ 8 mg/kg if ≥30 kg and 8 mg/kg or 10 mg/kg randomised if <30 kg). Pts were 2-17 y old with ≥5 active joints and >38°C fever for ≥5 days (sjia) or ≥3 of ≥5 joints with limited motion (pjia). Blood samples were analysed for TCZ, IL-6R blockade markers (IL-6, soluble IL-6R [sil-6R]), C-reactive protein (CRP) levels and erythrocyte sedimentation rate (ESR). Efficacy was measured as JIA ACR30/50/70/90 response rates. Population PK (poppk) modelling was used to further analyse serum TCZ concentration data in addition to descriptive summary statistics.

## Results

In sjia pts, mean serum TCZ concentrations over time and steady state TCZ exposures at wk 12 were similar between TCZ 8 mg/kg and 12 mg/kg BW groups. Predose concentrations trended upwards over time, stabilising by wks 10-12. IL-6, sil-6R, CRP and ESR profiles were overlaid between BW groups, showing similar IL-6R blockade. Consistently, primary efficacy outcomes by JIA ACR 70/90 response were also comparable. In pjia pts, the <30 kg group taking TCZ 8 mg/kg had lower TCZ concentrations than the <30 kg group taking 10 mg/kg and the ≥30 kg taking 8 mg/kg, which were similar to each other. IL-6, sil-6R, CRP and ESR profiles indicated reduced IL-6R blockade in pts <30 kg taking TCZ 8 mg/kg than in the other two groups. Consistent with this, quartile analysis showed lower JIA ACR30/50/70/90 response rates at wk 16 in the lowest TCZ exposure quartile. No clear trends in adverse events across exposure quartiles were seen in sjia or pjia pts. Poppk analysis showed similar linear clearance of TCZ in sjia and pjia pts, but the Michaelis-Menten constant in target-mediated clearance was 3-4× higher in sjia pts.

## Conclusion

Results support adjusting BW-based TCZ dosing in sjia/pjia pts with the optimal dosages: sjia, TCZ 12 mg/kg Q2W for BW<30 kg and 8 mg/kg Q2W for BW≥30 kg; pjia, TCZ 10 mg/kg Q4W for BW<30 kg and 8 mg/kg Q4W for BW≥30 kg. Taking into consideration comparable linear clearances in sjia/pjia pts, the higher Michaelis-Menten constant in target-mediated clearance for sjia pts may be due to higher cell surface IL-6R levels. Observations are consistent with higher baseline IL-6 signalling in sjia pts and potentially explain the need for higher dose regimens to saturate IL-6R for optimal therapy in sjia pts.

## Disclosure of interest

P. Lu Employee of: Roche, J. Hsu Employee of: Roche, C. Keane Employee of: Roche, S. Fettner Employee of: Roche, J. Wang Employee of: Roche, N. Ruperto Grant/Research Support from: Abbott, astrazeneca, BMS, Centocor, Lilly, Francesco Angelini, GSK, Italfarmaco, merckserono, Novartis, Pfizer, Regeneron, Roche, Sanofi Aventis, Schwarz Biosciences, Xoma, Wyeth, Consultant for: Abbott, astrazeneca, BMS, Centocor, Lilly, Francesco Angelini, GSK, Italfarmaco, merckserono, Novartis, Pfizer, Regeneron, Roche, Sanofi Aventis, Schwarz Biosciences, Xoma, Wyeth, Speakers Bureau: Abbott, Boehringer, BMS, Novartis, Astellas, Italfarmaco, medimmune, Pfizer, Roche, O. Harari Shareholder of: Roche, Employee of: Roche, H. I. Brunner Consultant for: Novartis, Genentech, medimmune, EMD Serono, AMS, Pfizer, UCB, Janssen, Speakers Bureau: Genentech, F. De Benedetti Grant/Research Support from: Abbott, Pfizer, BMS, Roche, Novimmune, Novartis, SOBI.

